# Examining the Associations of Altered Metabolism in Black Women with Endometrial Cancer

**DOI:** 10.1007/s40615-026-03177-7

**Published:** 2026-09-18

**Authors:** Ida Micaily, Norman G. Rosenblum, Rebeca Kelly

**Affiliations:** Sidney Kimmel Comprehensive Cancer Center at Thomas Jefferson University, 1025 Walnut Street, Suite 700, Philadelphia, Pennsylvania 19107 United States of America

**Keywords:** Disparities, Metabolism, Black, Endometrial cancer

## Abstract

Metabolic syndrome is a modifiable risk factor in endometrial cancer patients and is predictive of disease outcomes in endometrial cancer survivors. While metabolic syndrome has been on the rise in the United States, there is also a concerning, disproportionate increase in the prevalence of aggressive endometrial cancer subtypes in Black women and with worse clinical outcomes. The purpose of this narrative review is to summarize the evolving landscape of research and the complex intersectionality of diagnostic and therapeutic disparities and survivorship considerations, in Black women with endometrial cancer. The goal of this review is to bring attention to these disparities to improve gynecologic oncologic care for Black women. Reviewing the mechanisms, molecular and social determinants of disease in this population can provide a foundation for future improvements in research, interventions in prevention, detection, and treatment aiming at improving survival outcomes for Black women with endometrial cancer.

## Introduction

Endometrial cancer, a malignancy of the lining of the uterine cavity, is the most common gynecologic malignancy in the United States and a leading cause of cancer related deaths [1]. While endometrial cancer is the fourth most common malignancy among women, it is now the deadliest of all gynecologic cancers [1]. It is estimated that approximately 69,120 new cases of endometrial cancer were diagnosed in the United States in 2025, with about 13,860 of those resulting in death [1]. Alarmingly, both endometrial cancer incidence and mortality rates have been on the rise in the past decade, with a 1.7% yearly increase in the latter [2]. It is predicted that the incidence of endometrial cancer will continue to rise worldwide, as the incidence has grown over 20 times in all age groups [2]. Countries with vast socioeconomic transitions have seen a rapid increase in endometrial cancer prevalence [3]. Notably, there has also been a trend increase in more aggressive histological subtypes of endometrial cancer globally [2].

The median age of diagnosis for endometrial cancer is 60 years in all women, and endometrial cancer is more common in Black women than other racial and ethnic groups [1]. While the diagnosis of early-stage endometrial cancer often occurs in post-menopausal women, an increasing number of younger, reproductive age women are being diagnosed. In the United States, there are disproportionate incidence and mortality rates for Black women with endometrial cancer [2]. Mortality in Black women with endometrial cancer has surpassed that from ovarian cancer since 2005 [3]. Of all endometrial cancer cases, 10 to 15% of patients present with advanced-stage disease, with a 5-year survival rate of only 17% among those who present with distant metastasis [4]. Black women particularly have a disproportionate incidence (43%) of high-risk and aggressive histologic subtypes (i.e. serous, carcinosarcoma, clear cell, mucinous, undifferentiated/dedifferentiated), 26 per 100,000 person-years) [1, 3, 5–7]. Irrespective of histological subtype, the five-year overall survival is lower for Black women with endometrial cancer than White women [2].

A 270,000-patient cohort study for patients with endometrial cancer suggests significant survival differences in Black women that is likely attributed to a multifactorial multitude of reasons such as comorbidity score, neighborhood income, insurance status (for patients under the age of 65), histologic subtype, disease stage, treatment and unexplained factors [5]. Several studies review several compounding risk factors [5–16], including Doll et al.’s evaluation of structural racism, sexism, history of trauma and intersectionality of these and other subjects that directly relate to the survival differences in Black women [8]. The Society of Gynecologic Oncology’s (SGO) 2026 evidence-based review identifies genetic, population/environmental, and unexplained factors as an additional axis of endometrial cancer risk with direct relevance to Black women [6]. A dedicated analysis of endometrial cancer risk factors, specifically in Black women, further highlights that the risks affecting them cannot be assumed to mirror that described in predominantly White cohorts [17]. Population-level analyses further demonstrate that individuals residing in areas of high social vulnerability—as measured by socioeconomic status, housing, transportation, and demographic composition—are also more likely to be diagnosed with non-endometrioid than endometrioid histology [18]. Among environmental exposures, heavy and long-term use of chemical hair relaxers and straighteners has been associated with increased uterine cancer risk, as demonstrated in a prospective cohort drawn from the Black Women’s Health Study [6, 18, 19]. Additionally, geographic locations where there may be a lack of access to gynecologic oncologists, inadequate insurance coverage, and public policy holes that cause disparate health care gaps can all affect outcomes for Black women [12].

Altogether, there is an urgent unmet need to address the severe healthcare disparities that Black women face as Black women are developing endometrial cancer at a younger age, have more aggressive disease, and have a higher risk of recurrence and death. Given the increasing prevalence of metabolic syndrome and the aggressive molecular subtypes of endometrial cancer, the aim of this review is to survey the current landscape of literature in metabolic changes associated with endometrial cancer, particularly in Black women, and to highlight important areas for future research that may lead to diagnostic, treatment and survivorship needs in this population to improve outcomes.

## Metabolic Syndrome and Endometrial Cancer

The global rise of endometrial cancer has been associated with a surge in metabolic syndrome, which has been deemed the strongest risk factor in all histological subtypes of endometrial cancer [4]. Metabolic syndrome is defined as a group of associated conditions, including hypertension, high blood sugar, excess fat tissue around the waist and abnormal cholesterol. Prior studies have shown that while the lifetime endometrial cancer risk is only 3% in women with those categorized as having normal body-mass index (BMI), that risk increases by more than 50% with every 5-unit increase of BMI [4]. There is a growing interest in understanding and addressing metabolic syndrome, tumor metabolism and the treatment of endometrial cancer in Black women.

Metabolic syndrome is associated closely with obesity, which is defined as a body mass index (BMI) ≥ 30 kg/m2. Metabolic syndrome is a modifiable risk factor for many diseases and includes a combination of many factors - notably diet, environment and genetics. A BMI over 40 increases the risk of cancer to as high as 70%, and it is estimated that 1 in 5 of all cancers are related to altered metabolism [20, 21]. Additionally, outcomes in cancer are worse when associated with metabolic syndrome with mortality rates 62% higher in women with high BMI in comparison to individuals characterized with normal BMIs [20]. A high BMI has consistently been reported to be associated with a heightened risk of endometrial cancer, particularly among postmenopausal women because of high circulating estrogens due to the conversion of the androgen precursor, androstenedione, to estrone or endogenous estrogens in adipose tissue [21]. Decreasing circulating estrogen through medications including the treatment of metabolic syndrome is an unmet need and currently undergoing investigation in preclinical and clinical research.

Black women are disproportionately at a higher risk for metabolic-related diseases through mechanisms that are not completely understood. While a genetic basis for disparities in high BMI among ethnicities is still being investigated [13–16], there is evidence to suggest that the unique social and environmental factors many Black women face may be a contributing factor to these differences [13, 14, 20, 22]. For example, neighborhood-level socioeconomic environment appears to play a casual role in the development of high BMI in Black women [23]. Black women living in neighborhoods with high levels of segregation and poverty have less access to resources necessary to maintain adequate education on healthy nutrition and physical activity [10, 11]. Lower parental education has been found to be associated with metabolic syndrome in adulthood [3]. Crucially, areas of food deserts and poor access to nutritious foods are implicated to worse endometrial cancer outcomes [23].

## Metabolic Markers in Endometrial Cancer

The physiologic basis of metabolism is complex, and several metabolic factors have been implicated in the pathogenesis of endometrial cancer, including adiponectin, leptin and insulin and signaling pathways [22, 24–32]. Adiponectin, leptin and insulin are circulating proteins help regulate energy storage and metabolism. Variations in expression of these proteins have been linked to many conditions such as metabolic syndrome, insulin resistance and the development of endometrial cancer.

Adiponectin is a protein that is released from adipose tissue that inversely correlates with fat deposition and regulates insulin sensitization [22]. Adiponectin has been shown to regulate gluconeogenesis, glucose uptake, beta-oxidation, triglyceride clearance and insulin sensitivity. Adiponectin has been implicated in the pathogenesis of endometrial cancer through its effects on metabolism [22, 24]. In the uterus, adiponectin receptors are present both in normal and cancerous endometrial tissue, with levels of adiponectin being inversely correlated with endometrial cancer risk [22, 24]. The effect of adiponectin on metabolically active tumor cells and its role in tumor progression has been reported [24]. Several studies have suggested that Black women carry lower baseline levels of adiponectin, possibly serving as a connection between altered metabolism and the increased incidence of endometrial cancer in Black women [22, 24–28].

Leptin and insulin are two well-studied factors associated with metabolic syndrome and have been linked to more aggressive tumor biology in Black women with endometrial cancer [22, 24–32]. Altered metabolism produces a pro-inflammatory state, releasing signaling molecules such as cytokines into the body that can alter cellular responses. High leptin levels stimulate cancer growth and decrease the destruction of cancer cells in endometrial cancer cells [28–31]. In addition, leptin may have effect on the tumor microenvironment by stimulating secretion of interleukin-6 (IL-6) and tumor necrosis factor alpha (TNF-a) from macrophages, and interleukin 2 (IL-2) and interferon gamma (IFN-y) from T-cells [28–31]. There is an urgent need to study the role of altered metabolism and immune response, and its association to the effect of systemic therapies, including immunotherapy.

Alterations of the JAK/STAT and PI3K/AKT/mTOR pathways are often seen in endometrial cancer. Insulin is an activator of these pathways, which plays a pivotal role in cell growth, proliferation and survival [22, 24–32]. Additionally, PI3K/Akt/mTOR activation increases cellular glucose uptake and promotes a metabolic switch from mitochondrial respiration to aerobic glycolysis [22]. Both pathways favor tumor growth and may be a component of the pathogenesis of endometrial cancer in Black women; targeting of these pathways in Black women with endometrial cancer needs to be studied further. Additionally, hypothesis generating preclinical data suggests that the modification of glucose control may enhance immunotherapy effect which is also pertinent to patients with endometrial cancer [33]. Future research into metabolism-related biomarkers in endometrial cancer may identify novel research targets in these pathways in the future to improve outcomes in this patient population.

## Disparities in Access to Preventative Care

A layered combination of hormonal factors has been shown to modulate endometrial cancer risk among Black Women. In the realm of hormonal exposures, combined oral contraceptives (OCP) confer durable, dose-dependent protection against endometrial cancer [34]. In a meta-analysis including 27,276 women with endometrial cancer, each additional five years of OCP use was associated with a 24% reduction in risk, with protection persisting for several years after discontinuation [34]. Similarly, a study has shown that parity acts in a protective manner, with a 31% risk reduction of endometrial cancer among parous versus nulliparous women [35]. Breastfeeding also independently reduces endometrial cancer risk, with pooled data from the Epidemiology of Endometrial Cancer Consortium demonstrating an 11% risk reduction among women who previously breastfed, with a larger reduction in risk associated with longer duration of breastfeeding [36]. Unopposed estrogen exposure—whether endogenous, as in chronic anovulation, or exogenous, as in estrogen-only menopausal hormone therapy—remains one of the most potent risk factors for endometrial cancer, conferring a two- to twelve-fold increase in relative risk, a risk that can persist more than a decade after exposure ends and that is substantially attenuated by the concurrent use of a progestin [37]. These protective and risk-modifying exposures are directly relevant given an increasing number of endometrial cancer diagnosis in young patients at reproductive age and warrant further investigation.

From a protective perspective, the levonorgestrel-releasing intrauterine system (LNG-IUS) can function simultaneously as a contraceptive method, a preventive alternative, and as a fertility-sparing treatment. As a contraceptive and/or preventive method, intrauterine device (IUD) use has been associated with a substantially reduced risk of endometrial cancer, attributed to localized progestin delivery that induces endometrial decidualization and glandular atrophy [38]. For reproductive-age women with endometrial intraepithelial neoplasia (EIN, formerly atypical hyperplasia) or grade 1 endometrioid endometrial cancer who wish to preserve fertility and in which no myometrial invasion is noted on MRI, progestin therapy—oral agents, the LNG-IUS, or both—is now established as the cornerstone of fertility-sparing management, as recommended per the SGO’s 2026 evidence-based guidelines [39, 40]. A 12-month complete response rate of 86% has been observed with LNG-IUS, compared with 66% for oral progestins alone. Further, a prospective phase II trial demonstrated a 12-month response rate of 90.6% for EIN and 66.7% for grade 1 endometrial cancer with LNG-IUS monotherapy [39, 41]. Notably, the phase II randomized feMMe clinical trial evaluating fertility-sparing approaches in young women with endometrial hyperplasia or clinical stage 1, FIGO grade 1 endometrioid adenocarcinoma investigated the use of LNG-IUS alone in this population versus in combination with metformin or with a structured weight-loss intervention. After a 6-month interval, a complete response rate of 61% was observed in the IUD alone arm, versus 67% when combined with a weight-loss program and 57% when combined with Metformin [42]. LNG-IUS present as both a preventive and a fertility-sparing therapeutic option for patients, allowing reproductive-age women to defer or avoid hysterectomy while engaging in weight-management interventions.

Access to the LNG-IUS and other long-acting reversible contraceptives (LARCs), however, is not distributed equitably, and this disparity is inseparable from a documented history of reproductive coercion targeting Black women in the United States. Compulsory sterilization programs disproportionately targeted Black women through the mid-twentieth century, and Depo-Provera was administered to Black women and girls, including as minors, before the drug’s formal approval for contraceptive use, a pattern scholars have termed “sterilization racism"[43, 44]. Dorothy Roberts’ foundational analysis situates these practices within a longer history in which Black women’s fertility has been alternately punished, surveilled, and coerced rather than supported as an exercise of autonomy [45]. This history continues to inform contemporary medical mistrust and care-seeking behavior, converging in the structural racism and intergenerational trauma described by Doll et al., which have contributed to survival disparities in Black women with endometrial cancer [8]. Currently, the SGO guidelines explicitly recommend weight loss for patients who are characterized as overweight and obese and are undergoing fertility-sparing treatment in order to improve the likelihood of both disease response and subsequent pregnancy and live birth, supporting a link between metabolic syndrome and optimized reproductive goals in endometrial cancer patients focused on fertility preservation [40, 42].

These historical dynamics are reflected in present-day disparities in contraceptive access. In nationally representative data, Black women report lower rates of LARC use than White women (7% versus 9%) despite comparable or greater clinical need, and Black women are more likely than White women to report cost as a barrier to obtaining their preferred contraceptive method [46, 47]. Additionally, qualitative research documents that young women of color perceive providers as more likely to recommend or press for LARC placement while being less responsive to requests for its removal, raising concerns about directive or coercive counseling, which conflicts with reproductive autonomy [48]. Taken together, these findings describe a paradoxical disparity: the population that may derive the greatest preventive and fertility-sparing benefit from the LNG-IUS—given increasing rates of younger age at endometrial cancer diagnosis, the environmental and genetic risk factors described above, and the disproportionate burden of aggressive disease in Black women—may also face the greatest structural barriers to accessing it, or may reasonably approach its recommendation with distrust given this history. Closing this gap requires contraceptive counseling and education that is transparent about both the protective and therapeutic roles of the LNG-IUS and that is explicitly grounded in patient-centered and autonomy-preserving decision-making.

## Diagnostic Challenges of Endometrial Cancer in Black Patients

There is emerging data highlighting significant racial disparities in the diagnosis of endometrial cancer between Black and White women [49–53]. Guidelines fail to diagnose almost half of endometrial cancers in Black women. Recent data suggests that pelvic ultrasound assessment of endometrial thickness is not sufficient in triage of Black women at risk for endometrial cancer and tissue sampling is effective, challenging the previous triage paradigm [49–52]. This is due to known ultrasonographic differences in endometrial thickness in Black and White patients [52, 54, 55]. Endometrial thickness on ultrasound failed to appropriately identify the rate of uterine fibroids and aggressive histological subtypes in endometrial cancer in Black patients [49]. Doll et al.’s study highlights the current challenges regarding diagnosis in various patient subsets [49]. Studies therefore have recently elucidated that biopsy should be the gold standard in the context of postmenopausal bleeding [50–53]. It is thought that the under and inadequate evaluation of women of color largely contributes to the presentation of more aggressive types and advanced stages of endometrial cancer in these patients.

## Bridging the Gap: Strategies for Equitable Treatment Approaches

The current standard of care therapeutic options for patients with endometrial cancer is based on previous clinical trial data. This includes consideration of surgical approaches, radiation therapy and systemic cancer-directed therapies. It is well known that there are significant barriers to medical counseling for patients of color, and that Black women are particularly at risk for inadequate and/or incomplete counseling. Medical distrust and mistrust are interwoven with a history of discrimination and inadequate shared decision-making. This may contribute to Black women refusing standard of care therapies which could contribute to poorer oncologic outcomes [54].

Data has shown that Black women receive inadequate genetic counseling, which can also influence risk factors, cancer screening and health-related outcomes for patients and their families [54]. Additionally, it is well known that women of color are highly underrepresented in clinical trials due to a multitude of factors including distrust in the system due to historical and ongoing abuse, misrepresentation and exclusion [56]. As the field continues to develop to better address oncologic disparities, there are layers of gaps that need to be filled to provide adequate counseling and to offer the best care for Black women with endometrial cancer.

## Addressing Barriers in Endometrial Cancer Survivorship

Given that excess body weight and lifestyle choices can be modifiable, ongoing research aims to improve weight loss strategies in women with endometrial cancer [55, 57–61]. Lifestyle modifications, which can include dietary changes and physical activity to aid in weight loss, is increasingly recognized to potentially reduce risk of death from cancer or increase time before cancer recurrence [55, 57, 58]. Improvements in diet and exercise can address many of the quality of life and psychosocial concerns that cancer survivors face [57, 58]. Dietary changes and increased physical activity may also improve outcomes for other health conditions that cancer survivors may encounter later in life. Further research is ongoing to strengthen and improve the complexity of care to enhance outcomes for cancer survivors. A survey has suggested that nutritional and exercise guidance is significant to survivorship programs [59].

When considering behavioral change, it has been shown that comprehensive approach that encompasses physical and mental health is often most efficacious [60]. Although there are clear benefits to improving metabolic health following endometrial cancer treatment, adherence to dietary programs has variable success [58–64]. Unfortunately, traditional lifestyle programs that combine diet and exercise have been shown to be less efficacious for Black women, and engagement and acceptability of these programs is also poorer [60, 61]. Notably, Black women engaging in these types of interventions lose on average 50% less weight compared to White counterparts [59]. This may be the result, in part, of a larger context of many other unique demands and stressors that Black women face [58]. Therefore, it is important to acknowledge these considerations (e.g. high levels of stress, limited access to some healthy foods) when attempting to change behaviors and provide (1) a dietary prescription that fits individualized needs, and (2) behavioral support that facilitates goal attainment [58–64]. There is evidence for physical activity as a facilitator of survivorship [60, 61]. Future psychological resources for women with endometrial cancer should incorporate acceptance-based behavioral therapy and environmental change together, as this strategy has been successful in obtaining significant weight loss after 1 year of treatment compared to standard behavior therapy or behavior therapy with environmental change [61].

Currently, there are ongoing studies investigating various weight loss methods for endometrial cancer patients [61–65], including lifestyle modification, exercise, weight reduction and dietary modifications in survivors. Survey studies evaluating weight changes in Black endometrial cancer survivors have been performed [60, 61]. These also include anti-inflammatory diets, metabolic medications and fasting [33]. Research is ongoing in several preclinical studies and clinical trials to elucidate if weight loss could potentially lead to better health outcomes in overall survival, progression free survival as well as survivorship in endometrial cancer patients [33]. Particularly, research interventions with metabolism, associated biomarker and genomic studies associated with endometrial cancer pathogenesis in Black women are needed.

**Fig. 1:**
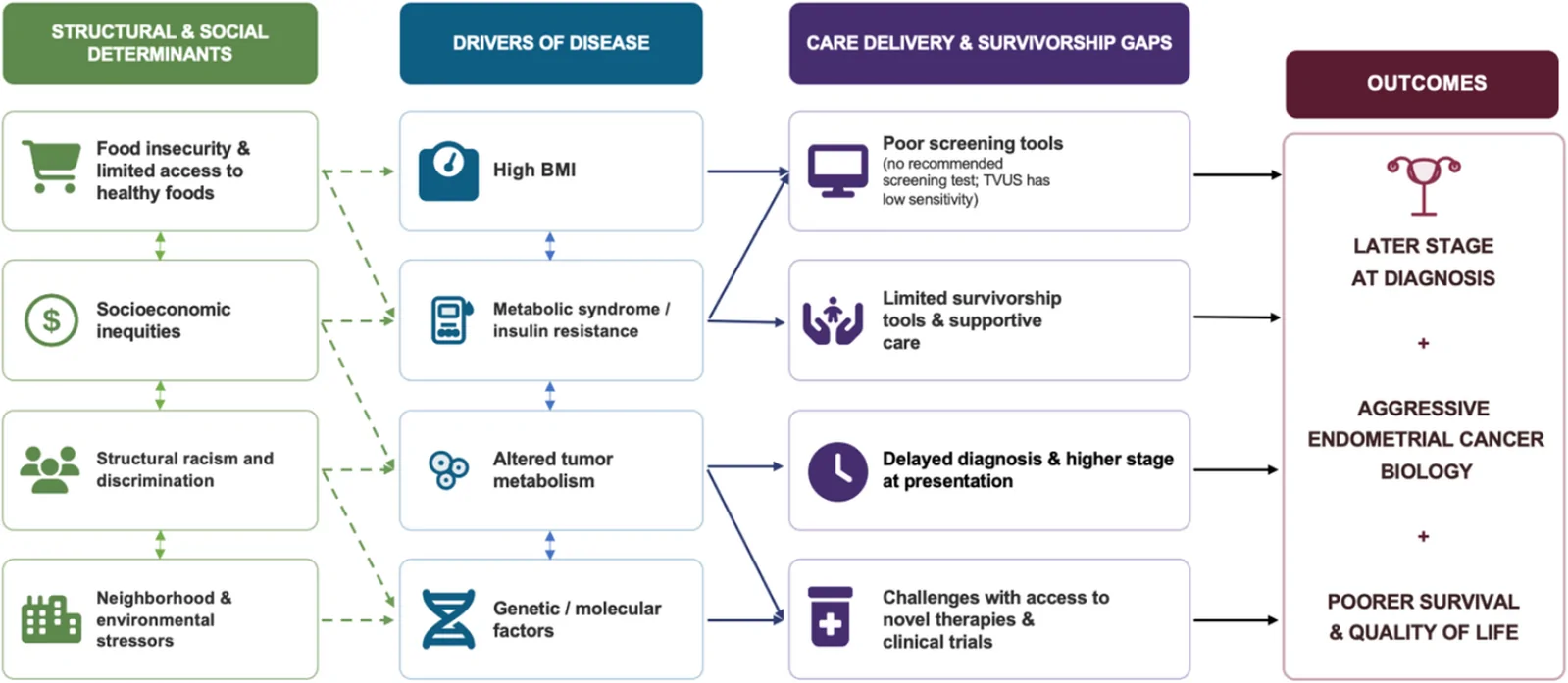
Factors in endometrial cancer care in black women that impact outcomes. Note: Fig. 1 is the created by the authors.

Important additional considerations of survivorship, particularly in Black women, is regarding supplementation of key nutrients such as vitamin D, as it has been stated that low vitamin D status can impact worsening survivorship [65]. In a single comprehensive cancer center study, it was found that patients were primarily interested in more guidance on supplement utilization in cancer survivorship [66]. However, another study indicated numerous barriers to patient participation in survivorship programs due to lack of access to transportation, financial constraints, health related issues, lack of knowledge of programs offered and other psychosocial barriers [67]. When discussing survivorship, it is also prudent to discuss financial toxicity of cancer directed therapy and acknowledging that this can affect our most vulnerable young patients of color significantly [68]. Survivorship care is complex and more funding is needed to improve disparities in survivorship programs so that Black women with endometrial cancer can improve their quality of life and long-term health related outcomes. (Fig. 1)

## Future Directions and Conclusion

While the primary aim of this review is to highlight ongoing research in metabolic, preventative, diagnostic, therapeutic and survivorship complexities and disparities in the care of Black women with endometrial cancer, we aim to highlight some of the dire unmet resource needs to improve outcomes. The evidence indicates that disparities in endometrial cancer risk and histology observed in Black women reflect a layered combination of genetic predisposition, environmental exposure, and structural social vulnerability, reinforcing the need for a better understanding of protective and access-related factors corroborating to such disparities.

First, access to adequate nutrition and the improvement of community services for Black women is pivotal, requiring research in policy changes to improve health access and engagement. While outside of the scope of this review, we aspire to encourage policy makers to provide funding support to focus on nutrition and education research to improve the understanding and treatment of metabolic syndrome in Black women. Second, further federal and institutional funding is pivotal in improving endometrial cancer research and access to care to focus on endometrial cancer prevention, enhanced diagnostic tools, and the impact of metabolism on cancer-directed therapy in Black women. In the era of precise and novel therapeutics, it is important to improve research in therapeutic disparities for enhanced treatment options for Black endometrial cancer patients. Third, more resources are needed by healthcare providers and institutions to improve survivorship resources to enhance quality of life for Black endometrial cancer survivors.

As this review is not exhaustive, it is important to highlight significant gaps so that a multidisciplinary approach is used to improve the lives of Black women. We must also emphasize that there is little to no data in other underrepresented communities, and significant work lies ahead and is necessary to address the complexity of needs to improve the care for all women. There is an urgent, unmet need to understand the mechanisms that drive disease processes in Black women living with endometrial cancer. Future research in uncovering genetic and molecular alterations associated with altered metabolism in endometrial cancer and evaluating the effects of lifestyle modifications that could prove instrumental in improving short term treatment-related and long-term survivorship-focused outcomes is urgently required.

## Data Availability

Data sharing not applicable to this article as no datasets were generated or analyzed during the current study.
